# The association between Major Depressive Disorder and Cannabis Use Disorder: a meta-analysis and meta-regression analysis

**DOI:** 10.1192/j.eurpsy.2025.923

**Published:** 2025-08-26

**Authors:** J. Alemar, J. M. Castaldelli-Maia, M. O. Pozzolo Pedro

**Affiliations:** 1 Faculdade de Medicina do ABC, Santo André, Brazil; 2 Neuroscience, Faculdade de Medicina do ABC, Santo André; 3 Psychiatry, Faculdade de Medicina da Universidade de São Paulo, São Paulo, Brazil

## Abstract

**Introduction:**

Major Depressive Disorder (MDD) is one of the most common psychiatric conditions, while cannabis is the most widely used illicit drug globally. Both MDD and Cannabis Use Disorder (CUD) hold significant epidemiological and health implications. Emerging evidence suggests a co-occurrence between cannabis abuse, dependence and depression, though studies remain limited.

**Objectives:**

To estimate the percentage of individuals with CUD who have comorbid MDD and those with MDD who have comorbid CUD.

**Methods:**

PubMed, SciELO, and Google Scholar were searched using keywords: ((abuse, cannabis[MeSH Terms]) OR (cannabis dependence[MeSH Terms])) AND ((depressive disorder, major[MeSH Terms]) OR (depressive disorder[MeSH Terms])). Original articles in English or Portuguese were included. Data collection followed PRISMA, MOOSE guidelines, and JBI critical appraisal. The final sample included 53 articles: 36 for the first meta-analysis and 17 for the second. A heterogeneity test (Q test) and "leave-one-out meta-analysis" were used. Prevalence rates were aggregated using random-effects models. Meta-regression and sensitivity analyses were conducted.

**Results:**

MDD showed a high prevalence among individuals with CUD, at 31.12% (95% CI: 25.71% to 36.80%). Prevalence was not significantly influenced by year, age, gender, population type, assessment period, region, or diagnostic criteria. CUD prevalence among those with MDD was 10.95% (95% CI: 7.08% to 15.53%), with higher rates in men and younger individuals. CUD prevalence appears to be increasing over time, though population type and assessment period did not significantly affect overall prevalence.

**Conclusions:**

This meta-analysis reveals a high prevalence of MDD among individuals with CUD and a significant prevalence of CUD among those with MDD, confirming a strong comorbidity. Cannabis use may exacerbate depressive symptoms, while those with MDD are at higher risk of developing CUD. Age, gender, and geographical factors influence this relationship. With increasing cannabis use, particularly among younger populations, the CUD-MDD comorbidity presents a growing public health issue. Further research is needed to explore the longitudinal link between these disorders.

**Disclosure of Interest:**

None Declared

